# Identification and Inhibition of the Druggable Allosteric Site of SARS-CoV-2 NSP10/NSP16 Methyltransferase through Computational Approaches

**DOI:** 10.3390/molecules27165241

**Published:** 2022-08-17

**Authors:** Shah Faisal, Syed Lal Badshah, Bibi Kubra, Mohamed Sharaf, Abdul-Hamid Emwas, Mariusz Jaremko, Mohnad Abdalla

**Affiliations:** 1 Department of Chemistry, Islamia College University Peshawar, Peshawar 25120, Pakistan; 2 Department of Biochemistry, Faculty of Agriculture, AL-Azhar University, Nasr City, Cairo 11751, Egypt; 3 Department of Biochemistry and Molecular Biology, College of Marine Life Sciences, Ocean University of China, Qingdao 266003, China; 4 Core Labs, King Abdullah University of Science and Technology (KAUST), Thuwal 23955-6900, Saudi Arabia; 5 Smart-Health Initiative (SHI) and Red Sea Research Center (RSRC), Division of Biological and Environmental Sciences and Engineering (BESE), King Abdullah University of Science and Technology (KAUST), Thuwal 23955-6900, Saudi Arabia; 6 Key Laboratory of Chemical Biology (Ministry of Education), Department of Pharmaceutics, School of Pharmaceutical Sciences, Cheeloo College of Medicine, Shandong University, 44 Cultural West Road, Jinan 250012, China

**Keywords:** COVID-19, methyl transferase, allosteric site, inhibitors, molecular docking, simulation

## Abstract

Since its emergence in early 2019, the respiratory infectious virus, SARS-CoV-2, has ravaged the health of millions of people globally and has affected almost every sphere of life. Many efforts are being made to combat the COVID-19 pandemic’s emerging and recurrent waves caused by its evolving and more infectious variants. As a result, novel and unexpected targets for SARS-CoV-2 have been considered for drug discovery. 2′-O-Methyltransferase (nsp10/nsp16) is a significant and appealing target in the SARS-CoV-2 life cycle because it protects viral RNA from the host degradative enzymes via a cap formation process. In this work, we propose prospective allosteric inhibitors that target the allosteric site, SARS-CoV-2 MTase. Four drug libraries containing ~119,483 compounds were screened against the allosteric site of SARS-CoV-2 MTase identified in our research. The identified best compounds exhibited robust molecular interactions and alloscore-score rankings with the allosteric site of SARS-CoV-2 MTase. Moreover, to further assess the dynamic stability of these compounds (CHEMBL2229121, ZINC000009464451, SPECS AK-91811684151, NCI-ID = 715319), a 100 ns molecular dynamics simulation, along with its holo-form, was performed to provide insights on the dynamic nature of these allosteric inhibitors at the allosteric site of the SARS-CoV-2 MTase. Additionally, investigations of MM-GBSA binding free energies revealed a good perspective for these allosteric inhibitor–enzyme complexes, indicating their robust antagonistic action on SARS-CoV-2 (nsp10/nsp16) methyltransferase. We conclude that these allosteric repressive agents should be further evaluated through investigational assessments in order to combat the proliferation of SARS-CoV-2.

## 1. Introduction

Allostery functions as a primary regulative mechanism in a wide range of biological processes, such as cell signaling, enzymatic catalysis, and other metabolic processes [1,2]. The ability of allosteric proteins to regulate the activity of orthosteric sites in response to a signal, which is mainly fueled by effectors’ attachment at separate, frequently distant functional sites, also known as allosteric sites, is central to their functionality [3,4]. Effector binding to an allosteric site allows a protein to transmit perturbations from an allosteric site to its active catalytic site, allowing for fine control of the protein’s core activities [5,6].

The allosteric sites in proteins have significantly less conserved residues compared to the active catalytic or orthosteric sites of proteins [7]. Similarly, the shapes of the orthosteric and allosteric sites in proteins are also different [8]. The amino acid residues present in these sites are also different; allosteric sites tend to have more hydrophobic residues, while orthosteric sites are rich in polar amino acids [9,10]. Molecules that target allosteric sites are also more diverse than the orthosteric ligands; allosteric modulators are more rigid and aromatic than the orthosteric ligands, which bind to the catalytic sites [11].

Several studies have previously reported that allosteric modulators have many important advantages over the orthosteric ligands that occupy a protein’s active site. Allosteric modulators have two advantages: high selectivity and few side effects [11,12,13]. It has been reported that several kinase enzymes and GPCRs have potent orthosteric ligands available that target its activity. However, orthosteric ligands for these intended GPCRs and protein kinases are known to have cross-reactivities with other homologous proteins, resulting in undesirable side effects and off-target toxicity [14,15]. Conversely, a compound that specifically binds to an allosteric site of this kinase enzyme is highly selective in targeting the other three closely related structures of this kinase enzyme rather than the orthosteric ligand [16]. Similarly, the NSP10/NSP16 Methyltransferase of SARS-CoV-2 is an essential and critical enzyme and performs vital functions in the SARS-CoV-2 viral life cycle [17,18]. The SARS-CoV-2 2′-o-methyltransferase (nsp16) protects viral RNA from cellular innate immunity by participating in the synthesis of a particular arrangement of N-methylated guanosine triphosphate and C2′-O-methyl-ribosyladenine at the 5′ end of the RNA molecule. The RNA cap is a structure that resembles the natural mRNA of the host cells, stabilizes the RNA, and ensures that it is translated effectively. The cap is formed when 5′-RNA triphosphatase removes a γ-phosphate from the nascent RNA’s 5′-triphosphate end. Guanylyl-transferase then adds a guanosine monophosphate (GMP) to the newly generated 5′-diphosphate end of the RNA [18]. Finally, NSP10/NSP16 methyltransferase and nsp14 enzymes perform two phases of methylation: nsp14 adds a methyl group at N-7 of the guanosine nucleobase (N-7 methyltransferase), and nsp16 adds a methyl group at C2′-O of the next nucleotide of the viral RNA [19]. This mechanism is critical for RNA stability, as it prevents the host enzymes from degrading it [20,21]. The NSP10/NSP16 methyltransferase of SARS-CoV-2 has a potent orthosteric inhibitor sinefungin available [17]. Sinefungin targets its active site, but it has been reported that this inhibitor can target the host methyltransferases which can result in off-target toxicity similar to that described in the above-discussed paragraphs, where targeting of GPCRs and kinase enzymes resulted in off-target toxicity due to the relatedness of the structures of these proteins [22,23,24]. Due to the limitations of sinefungin and its side effects, its use for targeting SARS-CoV-2 is limited. Therefore, targeting the allosteric site of this NSP10/NSP16 methyltransferase (MTase) can result in an efficient and highly selective allosteric repressor discovery that will help us in better controlling and mitigating the effects of SARS-CoV-2.

There are several approaches available to investigate the allostery in proteins. Some are experimental approaches, such as X-ray crystallography, cryo-electron microscopy (cryo-EM), and NMR spectroscopic techniques [13,25,26,27,28]; some are computational techniques. However, due to the complicated nature of allostery in biological processes, determining allosteric sites in proteins and the mechanism of allostery using experimental procedures, such as X-ray crystallography and NMR, is often time-consuming and frequently unsatisfactory [29,30]. In contrast, computational approaches for the study of allostery are a viable alternative to experimental methods. These theoretical computational approaches offer reliable tools which aid in drug design and have been the focus of rigorous research [31,32,33,34,35]. The allosteric database (ASD) [9,32,33], ASBench [36], Allofinder [37], and Allosite-Pro [38,39] are some of the main advances that have aided the development of computational approaches for predicting allosteric sites and their modulators. In the last few years, ASD and ASBench data have aided the development of a huge number of state-of-the-art computational approaches. Most importantly, many of the theoretical predictions have been validated by practical data, implying that computational methods are effective in structure-based drug development [9,30].

Because of the mechanism and importance of MTase enzyme RNA cap creation in SARS-CoV-2, it is a promising pharmacological target, and blocking it could result in effective COVID-19 infection management. We will use these structure-based drug development computational techniques to find allosteric sites and allosteric drugs that target this important MTase enzyme, which is vital for SARS-CoV-2 viral replication, as inhibiting it can impede its viral lifecycle.

## 2. Materials and Methods

### 2.1. Allosteric Site Identification

First, a protein PDB structure of the SARS-CoV-2 MTase with PDB identifier number 6XKM [40] was retrieved from the RCSB Protein Data Bank [41]. Then, for the identification of the allosteric site, it was uploaded to the Allosite-Pro server (http://mdl.shsmu.edu.cn/AST/) (accessed on 26 May 2022) [38]. For the re-validation of the allosteric site, which we identified via Allosite-Pro, the same PDB was then uploaded to the PASSer (https://passer.smu.edu/) (accessed on 26 May 2022) [42] allosteric site identification server. After the allosteric site validation in the SARS-CoV-2 MTase, we then proceeded to the identification of compounds that targeted this identified allosteric site.

### 2.2. Allosteric Drug-like Compound Identification

For allosteric drug-like compound identification against SARS-CoV-2 MTase, an online computational allosteric drug discovery platform, Allofinder (http://mdl.shsmu.edu.cn/ALF/) (accessed on 26 May 2022) [35], was utilized. AlloFinder is an allosteric computational tool with two functions: allosteric modulator screening and allosterome mapping. The allosteric modulator screening function is comprised of a group of approaches developed in the lab, mostly by the researchers that made the Allofinder, which integrates several computational algorithms for the successful identification of allosteric drug molecules against a target protein.

The workflow of Allofinder, as developed by its researchers and employed in the present study, is as follows: First, the SARS-CoV-2 MTase PDB retrieved earlier was uploaded to the Allofinder server. Allofinder contains multiple allosteric drug-like compound libraries (ZINC diversity, CHEMBL diversity, SPECS diversity, and NCI diversity drug libraries) [43,44,45]. These libraries collectively contain ~119,483 compounds and are specially curated by the researchers using the Allo-like filter [46,47]. These curated drug libraries contain only allosteric drug-like compounds that target allosteric sites in proteins. After uploading our target protein, SARS-CoV-2 MTase PDB, the integrated Allosite-Pro algorithm [38] in Allofinder identified the previously identified and validated allosteric site of our target protein. This allosteric site was then selected, and the computational pharmacophore pocket-based algorithm model [48,49] for the selected allosteric site was constructed by Allofinder, allowing unbound compounds in the library to be swiftly ruled out during the screening process. Then, each of the above-mentioned ligand libraries was screened individually via the built-in genetic algorithm from Auto Dock Vina [50]. For each of the molecules in the pharmacophore-filtered subset, this provided a conformational sampling of an ensemble of docked conformations against our target protein. Finally, the Alloscore algorithm [51] of Allofinder identified the optimal binding energy from each compound’s conformational ensemble, which resulted in the potential allosteric drug-like compounds specifically targeting the SARS-CoV-2 NSP10/NSP16 MTase allosteric site.

### 2.3. Molecular Dynamics Simulation and MM-GBSA Studies

Using the Schrodinger’s Desmond module, the ligand-protein complex or holo-protein was placed inside an orthorhombic box with periodic boundary conditions that were generated at least 10 Å away from the protein’s outer surface. It was then filled with the necessary quantity of water molecules, according to the system setup protocol. The MD computations were performed using the SPC model and the OPLS3 force field. By adding 0.10 mol/L sodium and chloride ions to the simulation panel, so that the isosmotic condition was preserved. A pre-defined equilibration protocol was carried out prior to the simulation. The standard Desmond protocol at constant pressure and temperature (NPT ensemble, 300 K, 1 bar) and the Berendsen coupling technique with one temperature group were used to equilibrate the system. The SHAKE algorithm was used to limit the length of hydrogen atom bonds. To describe long-range electrostatic interactions, the particle mesh Ewald (PME) summation approach was used. We performed a 100 ns simulation for the top leads along with the holo-form of the enzyme [33,52].

For validation of the stable compound’s binding and their binding energies, the MM/GBSA approach was used. The binding energy, determined by Prime MM/GBSA of Schrödinger Suite, shows an adequate estimation of binding energies/affinities. Prime’s MM/GBSA protocol combines OPLS-ff molecular mechanics energies, a VSGB polar solvation model (GSGB), and a nonpolar solvation expression (GNP) involving nonpolar solvent-accessible surface area (SASA) and Van der Waals interaction to determine the accurate binding free energy estimation of a protein–ligand complex [53]. The ADMET and drug-likeness studies were also predicted via SwissAdme (Swiss Institute of Bioinformatics, Lausanne, Switzerland) and ADMETlab2.0 (Central South University, Changsha, Hunan, China), and for the visualization of the protein–ligand interaction, Bovia DS studio was used in this study [54,55].

Researchers have previously used this strategy to identify robust allosteric inhibitors against a variety of targets, and it has been effectively deployed and experimentally confirmed, such as the discovery of 9-cis retinoic acid, Ifenprodil, and K116 (AH-034/11963955) allosteric inhibitors against thyroid hormone receptors (TRs), the GluN1b:GluN2B-NMDA receptor, and the STAT3 protein, respectively [37]. Aside from these mentioned allosteric inhibitors, computational techniques have been successful in identifying several more allosteric inhibitors against various protein targets [56,57,58].

## 3. Results and Discussions

**Figure 1:**
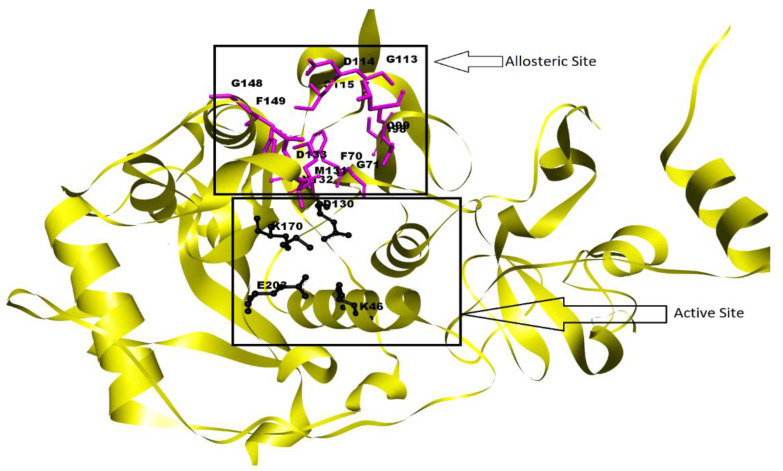
SARS-CoV-2 MTase active site residues are highlighted with black coloring, and allosteric site residues are highlighted with purple coloring; both are also labeled [17].

The SARS-CoV-2 NSP10/NSP16 MTase contains different binding pockets involved in the capping mechanism of the viral RNA of this virus. The S-Adenosyl-l-methionine (SAM) binding pocket of the MTase binds the respective compound, and through its catalytic tetrad (K-D-K-E), the amino acid residues in this pocket transfer a –methyl to the viral RNA for capping. This RNA is held in another binding pocket near the SAM binding pocket of this MTase where this methylation occurs [59]. Several researchers have indicated the presence of a cryptic allosteric site near the active site of the SARS-CoV-2 NSP10/NSP16 MTase [60]. Through Allosite-Pro [38] and PASSer [42] allosteric site identification servers, we identified an allosteric site in the SARS-CoV-2 NSP10/NSP16 MTase, and the analysis of this allosteric site in Pymol revealed the allosteric residues of this site. The allosteric residues of this pocket identified are F70, G71, S98, D99, L100, G113, D114, C115, M131, Y132, D133, G148, and F149, while the Active Site is composed of the following catalytic residues: K46, D130, K170, and E203. The active catalytic and allosteric site of the SARS-CoV-2 NSP10/NSP16 MTase can be seen in Figure 1, while other parameters of the allosteric site (i.e., pocket volume, SASA, perturbation score, and allosite score) are provided in Table 1.

As discussed above, the allosteric sites in proteins and the compounds that target these sites are relatively different from the orthosteric sites and the compounds targeting these sites. As the features and characteristics of the allosteric sites and allosteric inhibitors are different, the Allofinder server [37] utilizes the Alloscore [51] algorithm for ranking the top allosteric protein and ligand complexes for better evaluation and identification of these allosteric inhibitors against protein targets. Alloscore takes into account all the features of an allosteric compound and protein complex, evaluates it, and ranks each of the identified allosteric compounds based on the (1) Molecular interactions (i.e., Van der Waals (VDW) and H-bonding interactions), (2) the hydrophobic effects, and (3) the deformation effects, which involve entropic movements for atoms, both in the ligands and in the protein allosteric site that are engaged in interactions. Apart from these, geometric terms, such as shape and surface feature, that include the buried molecular-volume (VOL) and buried polar-surface areas are also taken into account. Based on all these attributes, the Alloscore algorithm gives an alloscore-score to each of the allosteric protein-ligand complexes. Using the aforementioned criterion, the best allosteric leads identified against the SARS-CoV-2 NSP10/NSP16 MTase from each chemical library are discussed in the subsequent sections.

### 3.1. SARS-CoV-2 NSP10/NSP16 MTase Prospective Allosteric Inhibitors, Identified from the ZINC Diversity Chemical Library

When the virtual screening process by the Allofinder for the target protein (SARS-CoV-2 MTase) was performed, it generated 100 top leads identified from the thousands of compounds in the library and, based on the Alloscore algorithm, ranked all 100 of the compound leads. Based on the observations from the previous allosteric inhibitor discoveries via the Allofinder Alloscore algorithm, a potential allosteric drug identified against a protein target is mainly ranked in the top 5–10 in the list of identified compounds.

**Figure 2:**
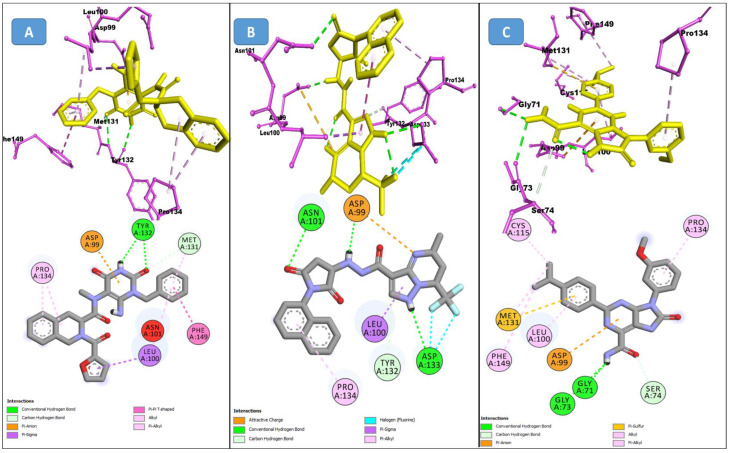
(**A**) represents the 3 (Upper panel) and 2-dimensional (Lower panel) interactive poses of ZINC000009464451, and (**B**,**C**) are the 3-dimensional representations of ZINC000002781694 and ZINC000004940454 with the SARS-CoV-2 MTase allosteric site respectively.

The Alloscore algorithm of Allofinder identified the ZINC000009464451 compound from the ZINC diversity library as the first hit. This compound had an alloscore of 7.49 and was on the first rank in the list of potential allosteric inhibitors against the SARS-CoV-2 NSP10/NSP16 MTase. This compound made several molecular interactions with the allosteric site residues. Different types of hydrogen bonding (conventional and carbon–hydrogen bonding) were seen with the TYR132 and MET131 residues during the analysis of their interactions. Moreover, Pi-sigma, alkyl, and Pi-alkyl, which are hydrophobic type interactions, were also present in large numbers between the allosteric site’s ASP99, PRO134, PHE149, LEU100, and MET131 amino acid residues and this identified lead compound. ZINC000002781694 and ZINC000004940454 were the second and third-best ranking compounds from the ZINC diversity library, with alloscore-scores of 7.29 and 7.19, respectively. Both of these compounds also showed strong molecular interactions with the target protein allosteric site. The presence of fluorine in the second compound further enhanced the molecular interactions by engaging twice with the ASP133 allosteric residue. Full 3-dimensional and 2-dimensional interactive poses can be seen in Figure 2, while Table 2 contains the chemical structures, molecular weights, and alloscore-score ranking of all the three identified compounds from the ZINC Library.

### 3.2. SARS-CoV-2 NSP10/NSP16 MTase Prospective Allosteric Inhibitors Identified from the CHEMBL Diversity Chemical Library

**Figure 3:**
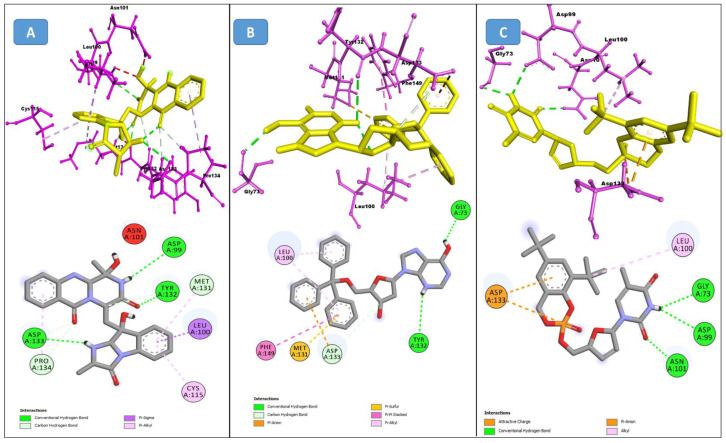
(**A**) Represents the 3- (Upper panel) and 2-dimensional (Lower panel) interactive poses of CHEMBL2229121, while (**B**,**C**) are the representations of CHEMBL209655 and CHEMBL319290 with the SARS-CoV-2 MTase allosteric site, respectively.

From the CHEMBL diversity library of compounds, Allofinder ranked CHEMBL2229121 as a first hit against the SARS-CoV-2 NSP10/NSP16 MTase with an alloscore-score of 7.5. This compound made multiple conventional and Carbon-hydrogen bonding molecular interactions with ASP99, ASP133, and TYR132 of the allosteric site, along with that Van der Waals type of interactions with the PRO134 and ASP133 were also present between them. Moreover, LEU100, CYS115, MET131, and PRO134 of the allosteric binding pocket exhibited strong hydrophobic interactions of Pi-sigma and Pi-alkyl type with the aromatic rings of this compound. The other two top-ranked compounds CHEMBL209655 and CHEMBL319290 had an alloscore-score of 7.35 and 7.23 respectively. These two compounds also showed strong molecular interactions of various types. These two compounds were also rich in aromatic moieties, which resulted in strong hydrophobic interactions between the allosteric site of SARS-CoV-2 NSP10/NSP16 MTase and these compounds. The second lead compound had strong Pi-Sulfur interactions with MET131, it also exhibited Pi-anion, Pi-alkyl type strong hydrophobic interactions and strong hydrogen bonding of multiple types with the CYS115, ASP114, LEU100 ASN101, and PHE149 of the allosteric site. The third compound also showed strong interactions of multiple types but the majority of those molecular interactions were of strong hydrophobic type interactions. A full 3-dimensional and 2-dimensional interactive poses can be seen in Figure 3 while Table 3 contains the chemical structures, molecular weights, alloscore-score rankings of all three identified compounds from the CHEMBL Library.

### 3.3. SARS-CoV-2 NSP10/NSP16 MTase Prospective Allosteric Inhibitors Identified from the SPECS Diversity Chemical Library

**Figure 4:**
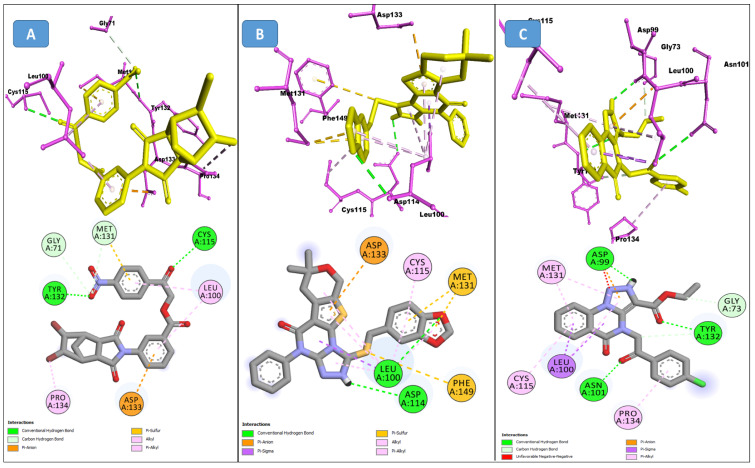
(**A**) Represents the 3- (Upper panel) and 2-dimensional (Lower pane) interactive poses of Specs_AK-918_11684151, while (**B**,**C**) are the representations of Specs_AJ-292_40706685 and Specs_AO-476_15578865 with the SARS-CoV-2 MTase allosteric site, respectively.

The SPECS diversity library of compounds has thousands of drug-like small molecules and strictly follows important drug-like rules, including Lipinski and Verber’s. The first identified best-ranked compound from this library was Specs_AK-918_11684151; it exhibited significantly high molecular contacts with the SARS-CoV-2 NSP10/NSP16 MTase allosteric site and made several types of interactions with it. The LEU100 and PRO134 exhibited strong hydrophobic interactions of the alkyl and Pi-alkyl types with this compound. A Pi-Sulfur molecular interaction of MET131 with the aromatic ring of this compound and a Van der Waals interaction with the –NO_2_ attached to this ring were also noted. Furthermore, the CYS115, ASP135, TYR132, and GLY71 of the allosteric site made several different conventional and carbon–hydrogen type hydrogen bonds, along with other types of interactions (i.e., Van der Waals and Pi-anion interactions), and the alloscore-score for this compound was 7.5. The other two best-ranked compounds, Specs_AJ-292_40706685 and Specs_AO-476_15578865, also showed strong and diverse molecular interactions, with alloscore-scores of 7.09 and 7.02, respectively. The second best-ranked compound exhibited significantly higher molecular interactions of a different nature with the LEU100 of the allosteric site and engaged this residue seven times via conventional H-bonding, alkyl, and Pi-alkyl hydrophobic type interactions. MET131 made two Pi-Sulfur molecular interactions, while ASP133 and 114, PHE149, and CYS115 also showed robust hydrogen bonding and stronger interactions of Pi-anion and Pi-sigma, along with the alkyl and Pi-alkyl hydrophobic type interactions. The third best-ranked compound also had higher molecular interactions and engaged LEU100 and ASP99 multiple times, along with the TYR132, PRO134, ASN101, ASP133, CYS115, GLY73, and MET131. The molecular interactions noted here between the SARS-CoV-2 NSP10/NSP16 MTase allosteric site and this compound were also diverse, such as the two other best-ranked compounds. Figure 4 shows the three compounds from the SPECS diversity library in full 3-dimensional and 2-dimensional interactive poses, while Table 4 shows their chemical structures, molecular weights, and alloscore-score rankings.

### 3.4. SARS-CoV-2 NSP10/NSP16 MTase Prospective Allosteric Inhibitors Identified from the NCI Diversity Chemical Library

**Figure 5:**
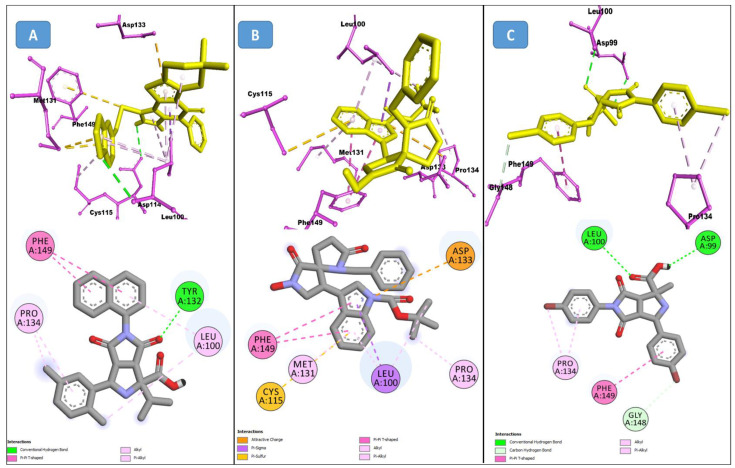
(**A**) Represents the 3- (Upper panel) and 2-dimensional (Lower panel) interactive poses of NCI-ID = 715319, while (**B**,**C**) are the respective representations of NCI-ID = 718571 and NCI-ID = 715313 with the SARS-CoV-2 MTase Allosteric site, respectively.

Several potent allosteric small molecule inhibitors targeting various flaviviruses essential enzyme NS2B-NS3 protease of ZIKV, DENV, and WNV were discovered by this technique from the NCI diversity library specifically targeting its allosteric site [55]. The allosteric compounds we identified here also showed robust molecular contacts with the SARS-CoV-2 NSP10/NSP16 MTase allosteric site. The first compound with NCI-ID = 715319 exhibited strong Pi-Pi T-shaped molecular interactions, engaging the PHE149 allosteric residue two times. Moreover, the TYR132 and ASN101 made conventional hydrogen bonds with this compound, along with PRO134 and LEU100 making strong alkyl and Pi-alkyl contacts with two of the –CHE and its two aromatic rings multiple times. The alloscore-score of this molecule was 7.36. The other two allosteric inhibitors, having identification number NCI-ID = 718571 and NCI-ID = 715313, also made strong molecular contacts with the target protein allosteric site and had alloscore-scores of 7.20 and 7.04, respectively. The second identified lead from the NCI library also exhibited the strong Pi-Pi T-shaped molecular interactions with the PHE149, along with Pi-sigma and Pi-Sulfur interactions of LEU100 and CYS115, respectively, while LEU100 also made Pi-alkyl contacts two times with this compound along with an alkyl contact of PRO134 allosteric site residue. The third compound from this library made multiple molecular interactions of various types. The LEU100, GLY148, and ASP99 of the allosteric site made multiple conventional and carbon–hydrogen bonding type interactions. The same allosteric residue of the SARS-CoV-2 NSP10/NSP16 MTase allosteric site, PHE148, that made the strong Pi-Pi T-shaped molecular interactions seen in the case of the other two identified leads, was also noted with this compound along with the PRO134 making Pi-alkyl and alkyl interactions with it, too. Figure 5 shows the three compounds from the SPECS diversity library in full 3-dimensional and 2-dimensional interactive poses, while Table 5 shows their chemical structures, molecular weights, and alloscore-score rankings.

## 4. Molecular Dynamics (MD) Stability Analysis Studies

### 4.1. MD Analysis of the SARS-CoV-2 MTase Holo-Form

**Figure 6:**
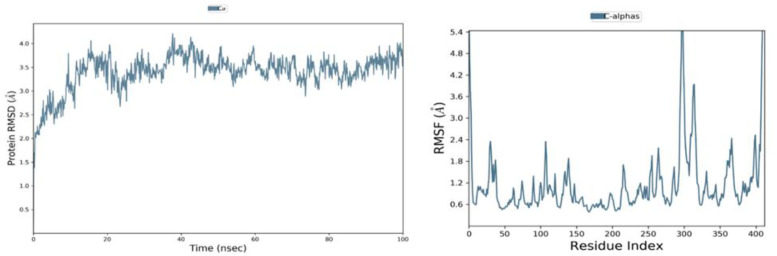
The C-α RMSD (Left hand side panel) and C-α RMSF (right hand side panel) of holo-enzyme.

**Figure 7:**
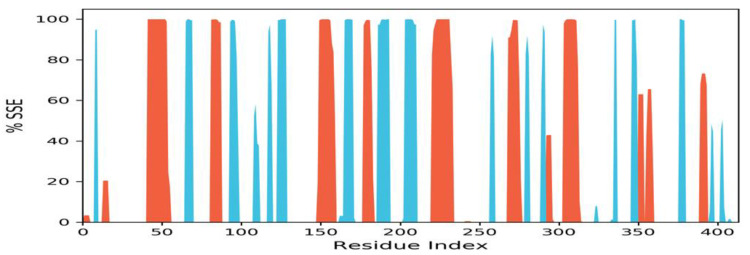
Secondary structure elements (SSE), white color—loops, red color—helix regions, turquoise color—strands in the protein.

The holo-form (un-liganded) SARS-CoV-2 MTase was simulated for 100 ns. Its RMSD graph is shown in Figure 6 (left side panel), where the protein C-alpha backbone did not fluctuate much and was stable throughout the simulation time. The RMSD fluctuation was at a range of 2 Å, which is fairly stable, and it remained like this in a stable form until the end. The RMSF values were also stable, but the protein’s termini’s regions fluctuated beyond 5 Å. The protein’s terminal regions’ residues move a lot and are more flexible than the other regions of the protein; a bit of a higher fluctuation at the 300th residue of this protein was also noted in the RMSF graph shown in Figure 6 (the right-hand side panel). The secondary structure element (SSE) analysis of this SARS-CoV-2 MTase protein revealed that it is rich in the loop regions (Figure 7, represented by white coloring). These loop regions connect secondary structure elements (SSE) (helix regions, β-strands, etc.) with each other. These loop regions often house the orthosteric sites of the enzymes as well. The protein was also rich in the beta-strand regions (turquoise color) and helical regions (red color). These regions provide stability to the overall structure of proteins.

### 4.2. MD Analysis of the SARS-CoV-2 NSP10/NSP16 MTase and CHEMBL2229121 Complex

**Figure 8:**
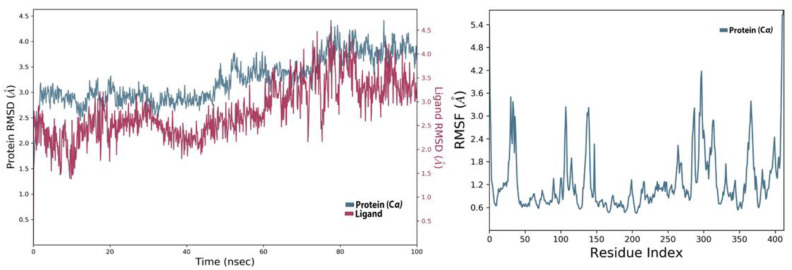
RMSD and RMSF of SARS-CoV-2 NSP10/NSP16 MTase and CHEMBL2229121 complex.

**Figure 9:**
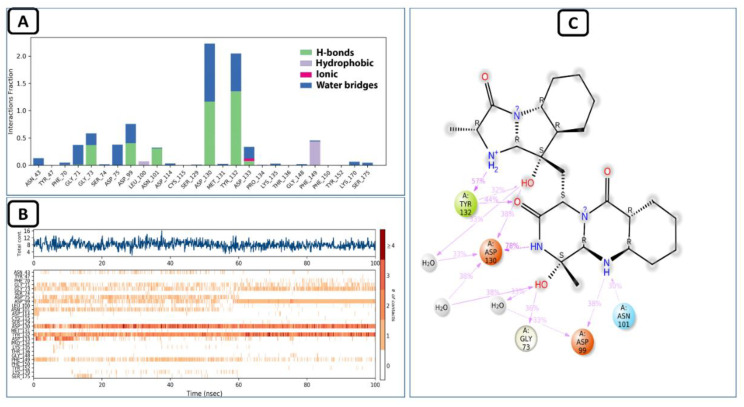
(**A**) Shows the protein-ligand interaction types, (**B**) shows the intensity of interactions, and (**C**) shows individual residues engaged by this ligand during the 100 ns simulation.

Molecular dynamics simulation studies of this complex revealed that it was stable during the 100 ns virtual simulation time. The RMSD and the RMSF values of the complex can be seen in Figure 8. The trajectory analysis of this complex for the interaction of this ligand with the allosteric site showed that this compound had various types of contacts during the 100 ns time. The water-bridged assisted hydrogen bond interactions were the most prominent, followed by the conventional hydrogen bonding of the allosteric ASP130 and TYR132 residues. The PHE149 also made hydrophobic interactions with the allosteric site of SARS-CoV-2 MTase, and the ASP99 and GLY73 allosteric residues also made hydrogen bond contacts with the allosteric site, as shown in the protein-ligand histogram diagram in Figure 9A. As seen in the Allofinder studies in the previous sections, this ligand retained and showed the same interactions in the MD studies. The intensity of each of the allosteric residues with this ligand is shown in Figure 9B,C, where GLY71, GLY73, ASP99, ASN101, ASP130, and TYR132 allosteric residues were in constant contact with the allosteric site of the SARS-CoV-2 MTase along the 100 ns simulation trajectory.

### 4.3. MD Analysis of the SARS-CoV-2 NSP10/NSP16 MTase and SPECS_AK-918_11684151 Complex

**Figure 10:**
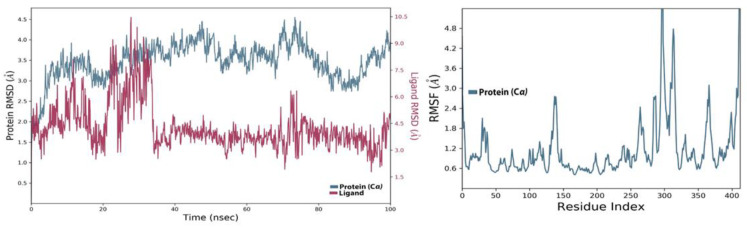
RMSD and RMSF of SARS-CoV-2 MTase and SPECS_AK-918_11684151 complex.

**Figure 11:**
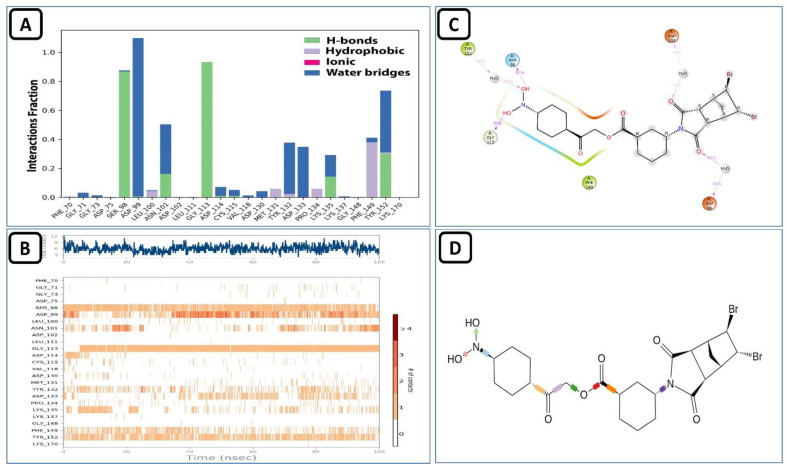
(**A**) Shows the protein-ligand interaction types, (**B**) shows the intensity of interactions, and (**C**) shows individual residues engaged by this ligand during the 100 ns simulation. (**D**) shows the ligands rotatable bonds or ligand torsions.

The ligand from the SPECS diversity library in the complex with the SARS-CoV-2 MTase allosteric site also remained stable during the simulation process. The RMSD of the protein in the complex can be seen in Figure 10. It started to fluctuate at 5 ns time, but the fluctuation was not too high and the change in RMSD was below the 3 Å threshold limit. The protein, RMSD, during the 100 ns simulation, remained like this in the stable form. The ligand, RMSD, also started low, but at about 20 ns time, the RMSD increased and remained like this until 35 ns. It came down after that, and the ligand then stabilized in the allosteric site. The increase of the ligand, RMSD, was due to the torsional movement of some of the movable bonds in this ligand. The interaction analysis of this ligand inside the allosteric site revealed that this compound was able to maintain the different types of molecular contacts previously seen in the Allo-finder studies. The protein-ligand histogram contact diagram can be seen in Figure 11A, where SPECS_AK-918_11684151 made hydrogen bonds of conventional type and those assisted by the water-bridged molecules present in the simulation medium. Hydrophobic contacts were also present in the histogram interaction diagram but the major molecular interactions seen in the MD simulation studies were H-bond type interactions. The intensity of the interactions along the simulation trajectory of the protein-ligand complex is shown in Figure 11B, while the individual interactions of this compound with the allosteric site residues can be seen in Figure 11C. The RMSF values of the protein were the same as seen in the holo-form of the protein. In Figure 11D, it can be seen that some of the bonds in this compound are colored. These colored bonds can move at certain angles in the allosteric site, which resulted in an increase in the ligand RMSD, at 2 ns time of the simulation.

### 4.4. MD Analysis of the SARS-CoV-2 NSP10/NSP16 MTase and ZINC000009464451 Complex

**Figure 12:**
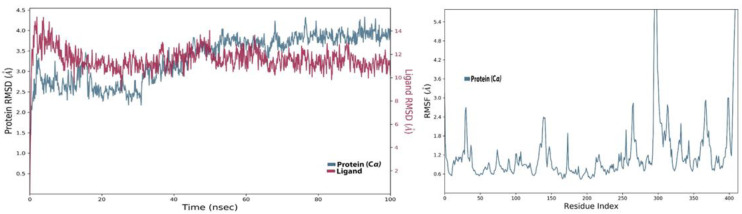
RMSD and RMSF of SARS-CoV-2 NSP10/NSP16 MTase and ZINC000009464451 complex.

**Figure 13:**
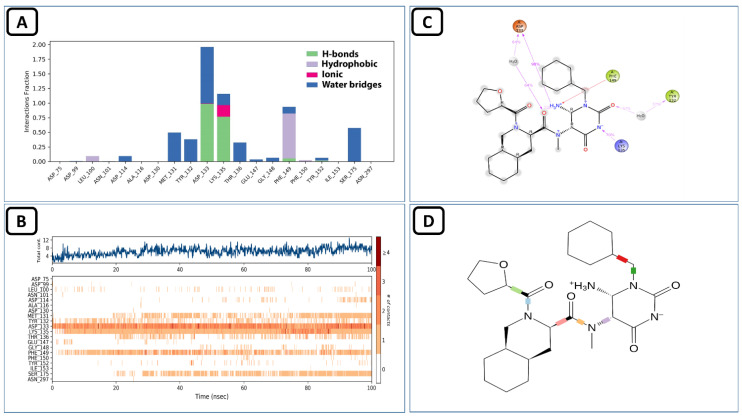
(**A**) Shows the protein-ligand interaction types, (**B**) shows the intensity of interactions, (**C**) shows individual residues engaged by this ligand during the 100 ns simulation, and (**D**) shows the ligand’s rotatable bonds or ligand torsions.

ZINC000009464451 and SARS-CoV-2 MTase complex remained stable. Its protein and ligand RMSD can be seen in Figure 12 aligned on the protein C-alpha backbone. The RMSF values also stayed the same, as seen in the holo-form of this protein discussed in the earlier section. The trajectory analysis for the interaction of this compound with the allosteric site revealed that it made multiple molecular contacts with the allosteric residues, as seen in the previous Allo-finder studies. The most prominent interactions during the 100 ns virtual simulation time noted were the hydrogen bonds made with the assistance of the water bridges present in the simulation medium. Hydrophobic interactions. along with the conventional type H-bonds were also present between this ligand and the SARS-CoV-2 MTase allosteric site (Figure 13A). The intensity analysis of these interactions during the MD simulation showed that the ligand remained buried in the allosteric site and an average of eight molecular contacts/ns was observed during the 100 ns total time (Figure 13B). The individual interactions of the allosteric residues and this compound are given in Figure 13C, while the ligand torsions that show the movements of certain bonds of this ligand are given in Figure 13D.

### 4.5. MD Analysis of the SARS-CoV-2 NSP10/NSP16 MTase and NCI-ID = 715319 Complex

**Figure 14:**
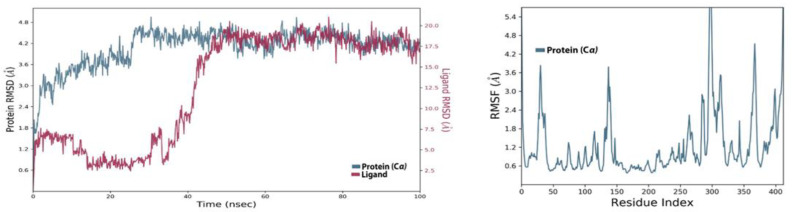
RMSD and RMSF of SARS-CoV-2 NSP10/NSP16 MTase and NCI-ID = 715319 1 complex.

**Figure 15:**
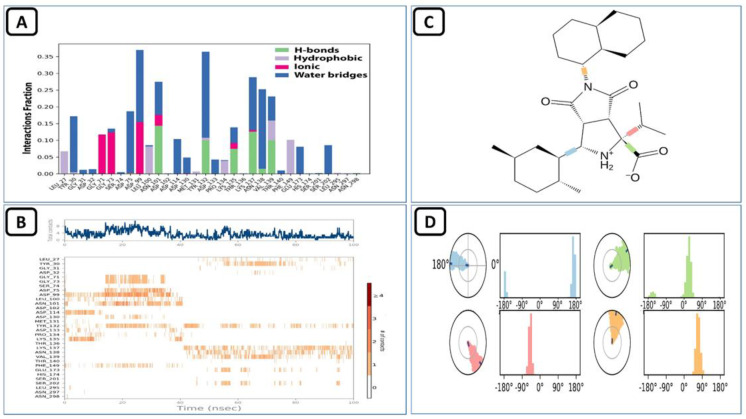
(**A**) Shows the protein-ligand interaction types, (**B**) shows the intensity of the interactions, (**C**) shows ligand torsions, and (**D**) shows the degree of the ligands rotatable bonds or ligand torsions on a radial plot and bar chart.

The compound, NCI-ID = 715319, identified from the NCI diversity library against the SARS-CoV-2 MTase at the start of molecular dynamics simulations, remained stable until the 35 ns mark. After that, the RMSD started to fluctuate too much inside the allosteric site (Figure 14). The RMSD later aligned on the C-alpha backbone of the protein; however, the trajectory analysis suggests that the ligand was in contact with the allosteric site of the SARS-CoV-2 MTase during the start of the simulation, but later, some major conformational changes occurred in the ligand structure that resulted in lower interactions. The interaction analysis of the trajectory revealed that this compound engaged in ionic, hydrophobic, and H-bond interactions with the allosteric site (Figure 15A), but its interaction intensity, as can be seen in Figure 15B, was low compared to the top identified leads from the other three compound libraries. The ligand torsional analysis diagram of the NCI-ID = 715319 (Figure 15C) shows the rotatable bonds, which are color-coded (Figure 15D) and provide information about the movement of some of the rotatable bonds in the ligand. As can be seen in the ligand torsional diagrams, one of the bonds (blue colored) had about 180° of rotation. The group attached to this bond is also bulky and might be the likely cause of the change in the ligand conformation inside the allosteric site.

## 5. MM-GBSA Binding Energy Studies

The relative energies of binding of the identified lead compounds from the four compound libraries with the allosteric site of the SARS-CoV-2 MTase were determined using the prime MM-GSBA methodology. It provided a variety of protein and allosteric ligand interactions, including Van der Waals, hydrophobic, and coulombic interactions, among others. The MM-GBSA results showed that these allosteric inhibitors have strong binding energies with the allosteric site SARS-CoV-2 MTase, and the binding energy terms (i.e., Van der Waals binding energy (dG Bind vdW), lipophilic (dG Bind Lipo), and coulomb energy (dG bind coulomb)) had lower negative values which correspond to stronger binding of the compounds to the allosteric site. Compounds CHEMBL2229121, ZINC000009464451, and SPECS AK-918 11684151 showed highly favorable ligand binding, whereas NCI-ID = 715319 from the NCI drug library showed relatively weak binding compared to these three lead compounds in the MM-GBSA studies.

The equation (MMGBSA dG Bind = Complex − Receptor − Ligand) is the energy of binding, where the ligand and receptor conformational changes are accounted for in the energy terms (Table 6). In the equation (MMGBSA dG Bind(NS) = Complex − Receptor(from optimized complex) − Ligand(from optimized complex)), “NS” stands for “no strain” and refers to the binding/interaction energy before taking into consideration the conformational changes required to form the complex between the receptor and the ligand (Table 7).

## 6. ADMET and Drug-Likeness Properties

The identified allosteric inhibitory compounds were evaluated for their pharmacokinetics and drug-likeness properties through the SwissADME web server. The Log S (ESOL), which are the estimated solubility values, were calculated, and all these compounds were listed as soluble. These compounds were absorbable through the GI tract and also had good lipophilicity values (logP values greater than 1 or less than 4 are ideal for a compound to be developed as a drug), which suggests that they are more likely to have ideal physicochemical and ADME qualities if utilized as oral medicines. The bioavailability score determines how likely a chemical is to be developed as an oral medication candidate. Typically, a bioavailability score of at least 0.10 is necessary to be considered a candidate; however, all of the compounds in this study had scores of 0.55, indicating that they have promising futures as medications. Except for the compound from the SPECS chemical library, which has one violation of the Lipinski rule due to the slightly high molecular weight, all other compounds completely followed this drug rule. None of these compounds inhibited the CYP1A2 enzyme. Except for the compound from the SPECS library, all other compounds were non-carcinogenic. They were also all non-AMES toxic, showed no toxicity in the Rat Oral Acute Toxicity model, and were not listed as hERG blockers, as predicted by the ADMETLab2.0. All of the ADME and drug-likeness values are provided in Table 8.

## 7. Sources and Data for the Identified Compounds

CHEMBL2229121 from the CHEMBL library has a common name (3-Hydroxyfumiquinazoline A) and is a phytochemical isolated from the aspergillus fumigatus fungus. The compound from the ZINC library, ZINC000009464451, has an IUPAC name, (3R)-N-(6-amino-1-benzyl-2,4-dioxopyrimidin-5-yl)-2-(furan-2-carbonyl)-N-methyl-3,4-dihydro-1H-isoquinoline-3-carboxamide. There is no source for this compound available, but it is available and its sample can be bought from a chemical compound vendor (https://www.enaminestore.com/catalog/Z28688115, accessed on 25 May 2022). The third compound, which is AK-91811684151, has an IUPAC name, 2-(4-nitrophenyl)-2-oxoethyl 3-((3aR,4R,5R,6R,7S,7aS)-5,6-dibromo-1,3-dioxooctahydro-2H-4,7-methanoisoindol-2-yl)benzoate. NCI-ID= 715319 is a pyrrole carboxylic acid derivative, and these compounds have been reported to be active against various biological targets.

## 8. Conclusions

By using the well-established computer-aided drug discovery and design (CADD) techniques, in our current investigations, we used several (CADD) tools to identify the allosteric site in SARS-CoV-2 methyltransferase and its prospective allosteric repressive compounds. We employed the Allo-finder allosteric drug discovery platform and its integrated multiple algorithms (i.e., Allo-site Pro algorithm, Autodock Vina genetic algorithm, Allo-Score algorithm, etc.) discussed in the materials and methods section and identified robust allosteric inhibitors against SARS-CoV-2 MTase. These allosteric inhibitors (CHEMBL2229121, ZINC000009464451, SPECS AK-91811684151, NCI-ID = 715319) exhibited robust molecular contacts and associations with the allosteric site of SARS-CoV-2 MTase. These compounds exhibited superior Allo-score rankings and showed multiple types of interactions (i.e., hydrophobic, Van der Waals, H-bonding, etc.) with the allosteric site residues. Additionally, the molecular dynamics studies showed that these compounds have constant, sustained, and stable interactions with the allosteric site of SARS-CoV-2 MTase, while the MM-GBSA energy calculations also showed strong binding of these compounds to this allosteric site. The results of the MM/GBSA analysis and molecular dynamics simulations suggest a potential inhibitory effect of these allosteric repressive drugs against SARS-CoV-2 methyltransferase. This study provided firsthand information about the allosteric site in an important enzyme of the SARS-CoV-2. It provided a base for targeting this enzyme, and its inhibition would make the virus RNA vulnerable to degradation by the host enzymes. The main limitation of these predicted studies is that they need to be evaluated and confirmed through various in vitro and in vivo studies to establish the role of these inhibitors against the SARS-CoV-2 methyltransferase. If they are found to inhibit the target enzyme, their toxicity studies will support their usefulness. In conclusion, we propose that *in vitro* and *in vivo* investigations can be conducted to further study, our identified potential allosteric repressive agents against SARS-CoV-2 in order to counter its spread.

## Figures and Tables

**Table 1:** SARS-CoV-2 MTase allosteric site parameters.

SN	Parameters	Score/Value
1	Allosite Score	0.580
2	Pocket Volume	517.96 (Å^3^)
3	SASA	323.947
4	Perturbation Score	0.295

**Table 2:** Chemical structures, compound IDs, their Molecular weights and alloscore-score ranking from ZINC Diversity Library.

S.N	Chemical Structure	Compound ID	Alloscore-Score	M.Wt
1	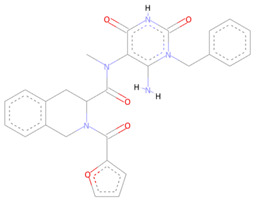	ZINC000009464451	7.49	477.3
2	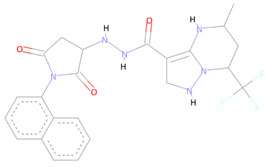	ZINC000002781694	7.29	469.3
3	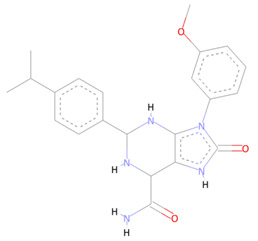	ZINC000004940454	7.19	387.3

**Table 3:** Chemical structures, compound IDs, molecular weights, and alloscore-score rankings from the CHEMBL Diversity Library.

S.N	Chemical Structure	Compound ID	Alloscore-Score	M.Wt
1	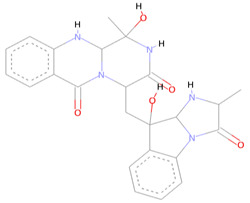	CHEMBL2229121	7.50	443.3
2	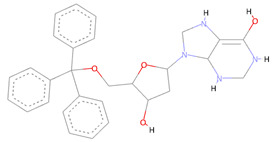	CHEMBL209655	7.35	473.3
3	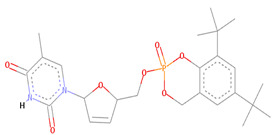	CHEMBL319290	7.23	472.2

**Table 4:** Chemical structures, compound IDs, molecular weights, and alloscore-score rankings from the SPECS diversity library.

S.N	Chemical Structure	Compound ID	Alloscore-Score	M.Wt
1	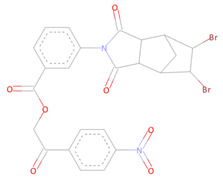	Specs_AK-918_11684151	7.50	588.1
2	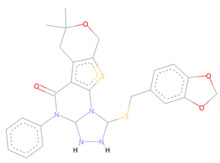	Specs_AJ-292_40706685	7.09	498.4
3	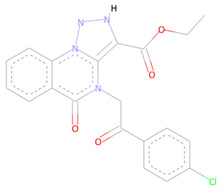	Specs_AO-476_15578865	7.02	396.7

**Table 5:** Chemical structures, compound IDs, molecular weights, and alloscore-score rankings from the NCI diversity library.

S.N	Chemical Structure	Compound ID	Alloscore-Score	M.Wt
1	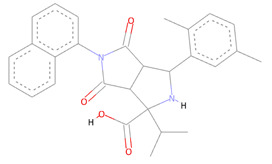	NCI-ID = 715319	7.36	430.3
2	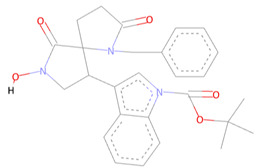	NCI-ID = 718571	7.20	447.3
3	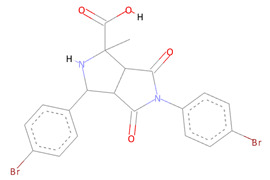	NCI-ID = 715313	7.04	494

**Table 6:** MM-GBSA binding energies (kcal/mol) with ligand-receptor conformational changes accounted for in energy terms.

S.No	Compound IDs	MMGBSA dG Bind	MMGBSA dG Bind Coulomb	MMGBSA dG Bind Hbond	MMGBSA dG Bind Lipo	MMGBSA dG Bind vdW
1	CHEMBL2229121	−63.35	−14.56	−2.72	−19.17	−56.28
2	ZINC000009464451	−38.27	33.50	−2.02	−15.10	−42.48
3	SPECS AK-91811684151	−54.77	−16.06	−1.74	−15.14	−47.23
4	NCI-ID = 715319	−29.24	15.9	−0.53	−8.60	−24.38

**Table 7:** MM-GBSA binding energies (kcal/mol) with ligand-receptor conformational changes not accounted for in energy terms.

S.No	Compound ID	MMGBSA dG Bind(NS)	MMGBSA dG Bind(NS) Coulomb	MMGBSA dG Bind(NS) Hbond	MMGBSA dG Bind(NS) Lipo	MMGBSA dG Bind(NS) vdW
1	CHEMBL2229121	−68.68	−17.04	−2.72	−19.38	−56.56
2	ZINC000009464451	−40.32	32.75	−2.02	−15.11	−44.01
3	SPECS AK-91811684151	−57.87	−16.9	−1.74	−15.04	−48.76
4	NCI-ID = 715319	−29.84	15.83	−0.53	−8.71	−24.64

**Table 8:** ADMET and drug-likness profile of the identified compounds.

SN	Compound Name	Log S (ESOL)	GI-Asorption	Lipinski Rule	Log Po/w (iLOGP)	Bioavailability Score	CYP1A2 Inhibitor
1	ZINC000009464451	−4.16/Moderately soluble	High	No Violations	2.67	0.55	No
2	CHEMBL222912	−2.98/Soluble	Low	No Violations	2.75	0.55	No
3	Specs_AK-918_11684151	−5.87/Moderately soluble	Low	1-Violation	3.21	0.55	No
4	NCI-ID = 715319	−4.10/Moderately soluble	High	No Violations	3.25	0.55	No

## Data Availability

Data can be requested from the first and principle authors through their emails.
